# Phone-Based Interventions in Adolescent Psychiatry: A Perspective and Proof of Concept Pilot Study With a Focus on Depression and Autism

**DOI:** 10.2196/resprot.7245

**Published:** 2017-06-16

**Authors:** Robert Yuzen Chen, Jordan Robert Feltes, William Shun Tzeng, Zoe Yunzhu Lu, Michael Pan, Nan Zhao, Rebecca Talkin, Kavon Javaherian, Anne Glowinski, Will Ross

**Affiliations:** ^1^ Washington University School of Medicine St. Louis, MO United States; ^2^ Saint Louis University School of Medicine St. Louis, MO United States; ^3^ Washington University School of Medicine Department of Psychiatry Division of Child and Adolescent Psychiatry St. Louis, MO United States

**Keywords:** telemedicine, depression, autistic disorder, mobile applications, text messaging, child, mental health

## Abstract

**Background:**

Telemedicine has emerged as an innovative platform to diagnose and treat psychiatric disorders in a cost-effective fashion. Previous studies have laid the functional framework for monitoring and treating child psychiatric disorders electronically using videoconferencing, mobile phones (smartphones), and Web-based apps. However, phone call and text message (short message service, SMS) interventions in adolescent psychiatry are less studied than other electronic platforms. Further investigations on the development of these interventions are needed.

**Objective:**

The aim of this paper was to explore the utility of text message interventions in adolescent psychiatry and describe a user feedback-driven iterative design process for text message systems.

**Methods:**

We developed automated text message interventions using a platform for both depression (EpxDepression) and autism spectrum disorder (ASD; EpxAutism) and conducted 2 pilot studies for each intervention (N=3 and N=6, respectively). The interventions were prescribed by and accessible to the patients’ healthcare providers. EpxDepression and EpxAutism utilized an automated system to triage patients into 1 of 3 risk categories based on their text responses and alerted providers directly via phone and an online interface when patients met provider-specified risk criteria. Rapid text-based feedback from participants and interviews with providers allowed for quick iterative cycles to improve interventions.

**Results:**

Patients using EpxDepression had high weekly response rates (100% over 2 to 4 months), but exhibited message fatigue with daily prompts with mean (SD) overall response rates of 66.3% (21.6%) and 64.7% (8.2%) for mood and sleep questionnaires, respectively. In contrast, parents using EpxAutism displayed both high weekly and overall response rates (100% and 85%, respectively, over 1 to 4 months) that did not decay significantly with time. Monthly participant feedback surveys for EpxDepression (7 surveys) and EpxAutism (18 surveys) preliminarily indicated that for both interventions, daily messages constituted the “perfect amount” of contact and that EpxAutism, but not EpxDepression, improved patient communication with providers. Notably, EpxDepression detected thoughts of self-harm in patients before their case managers or caregivers were aware of such ideation.

**Conclusions:**

Text-message interventions in adolescent psychiatry can provide a cost-effective and engaging method to track symptoms, behavior, and ideation over time. Following the collection of pilot data and feedback from providers and patients, larger studies are already underway to validate the clinical utility of EpxDepression and EpxAutism.

**Trial Registration:**

Clinicaltrials.gov NCT03002311; https://clinicaltrials.gov/ct2/show/NCT03002311 (Archived by WebCite at http://www.webcitation.org/6qQtlCIS0)

## Introduction

Psychiatric disorders in adolescent populations are extremely common, with a lifetime prevalence estimated up to 46.3% among individuals 13 to 18 years old [1]. As such, mental health disorders among US children have an estimated total annual cost of US $247 billion, with adolescents comprising the majority of these costs [2]. With the advent of mobile technology over the past two decades, there is an increasing need for innovative solutions to deliver inexpensive mental healthcare to our adolescent populations [3,4]. Telepsychiatry is a rapidly evolving field that promises to bridge evidence-based medicine with populations traditionally unable to connect with standard of care [5,6]. While videoconferencing remains the predominant evidence-based platform in telemedicine, phone-based interventions are another accessible and cost-effective method to target a variety of mental health conditions [7]. Here, we discussed the development of provider-prescribed automated text message (short message service, SMS) and phone call interventions for adolescent psychiatric disorders and their potential for improving patient outcomes.

### Adolescent Depression

Unipolar major depression (major depressive disorder, MDD) is a psychiatric disorder in which patients suffer at least one major depressive episode that causes significant distress or impairment in daily function, not attributable to mania or other causes [8]. In children between the ages of 10 and 19 years, the 12-month prevalence of major depression is estimated to be 7.5%, increasing significantly through adolescence and rising more quickly than in adults [2,9]. While the prevalence of depressive disorders increases with age for both sexes, adolescent females report a higher average percentage (25%) of depressive symptoms than their male (10%) counterparts [9-12]. Adolescents with depression exhibit decreased productivity in school leading to higher dropout rates, increased anxiety and anger, increased risk of developing other psychiatric and medical illnesses such as substance use disorder, and dramatically increased rates of suicide [13-19]. Currently, the main treatment options for adolescent depression are pharmacotherapy, psychotherapy, or a combination of both [20]. However, due to a high rate of non-compliance and non-responders, continual monitoring is critical to ensure optimal medication titration, decreased suicide rates, remission, and complete functional recovery [21-23]. Because underdiagnosis and undertreatment are common problems in adolescent depression, an annual depression screen using the 9-question Patient Health Questionnaire-9 (PHQ-9) or 2-question PHQ-2 is recommended for all adolescents beginning at the age of 12 until they are 21 [24-26].

Digital platforms are being explored both for screening adolescents at risk for major depression as well as for treatment of major depression in order to minimize costs, improve treatment adherence, optimize pharmacological titration, better track and manage symptoms, side effects, suicidality, and reduce behaviors that increase the risk of relapse. For example, a number of promising computerized cognitive behavioral therapy (cCBT) programs have been implemented in school settings with consistent success [23,24,27-30]. Another example—at-home depression questionnaires administered over mobile devices—have been used to remotely monitor clinically relevant metrics such as the PHQ-9 with remarkable sensitivity and specificity and little variability across race, gender, and age [31,32].

Intriguingly, telemedicine may be uniquely positioned to help depressive adolescents who have consistently responded positively to telemedicine interventions [33-35]. Because depressive adolescents are often reluctant to seek help from social networks, text-based interventions and crisis hotlines have emerged as highly utilized platforms for patients [36]. Adolescents often prefer the convenience, discreteness, increased communication effectiveness, and reduced anxiety associated with texting or talking on the phone compared with in-person evaluations with a physician [37]. Lublin and colleagues at DoSomething, an organization which uses texting to recruit volunteers for social advocacy programs, founded Crisis Text Line in 2013 after seeing an increasing number of teens using the text messaging system to report emotional crises and seek support [38]. Crisis Text Line is now one of the nation’s largest crisis text-based hotlines and has processed over 28 million texts to date [39]. As such, text message interventions present a unique intersection between adoptability and utility for detecting depression and even suicidality among adolescents.

### Adolescent Autism Spectrum Disorder

Autism spectrum disorder (ASD) is a neurodevelopmental disorder characterized by deficits in social communication and social interaction and the presence of restricted and repetitive patterns of behavior, interests, and activities [8]. Diagnosis, behavioral treatment, and monitoring children with ASD are still in its infancy. Managing ASD is time-intensive and costly and primarily consists of cognitive behavior therapy (CBT) interventions that target development and learning, social skills, and reducing maladaptive behaviors [40]. Although medications are commonly prescribed, they mainly target comorbid conditions and control symptoms, but do not provide treatment for underlying deficits [41]. Importantly, treatment for ASD is most effective when initiated early on and requires frequent follow-up adjustment cycles to be effective. This creates major barriers to care including time, cost, delayed diagnosis, access to specialists, and treatment adherence [42,43]. Aside from questionnaires taken during appointments (which are subject to recall bias), there are limited ways to monitor patient behaviors longitudinally between physician visits [44,45]. As such, there is a critical need to develop inexpensive methods to identify and monitor adolescents with ASD.

Digital platforms have emerged as new ways to improve access to care and better monitor adolescents with ASD. Most interventions have focused predominantly on mobile apps and videoconference services. Services that have been provided remotely through videoconference platforms include: primary evaluations for patients with ASD [46,47], cognitive behavioral interventions [48], and training healthcare professionals in rural areas to provide interventions to children with ASD [49]. Telemedicine systems that provide patients who live in rural areas with access to diagnostics and cCBT are capable of delivering medical care at dramatically reduced costs, without sacrificing diagnostic accuracy, quality, or patient satisfaction [50,51]. Mobile apps are another method for delivering services to children with ASD and their families remotely and electronically [52]. Because mobile apps are point-of-care, physicians can track patients’ symptoms with astounding temporal resolution. While these apps have developed more recently than video-based telehealth interventions, apps for surveying high-functioning ASD patients in real-time [53], teaching parents about ASD and treatment options [42], and social skills-building programs [54] already exist.

The benefits of electronic apps are wide-ranging. Enabled by portable computers and mobile devices, sampling methods such as ecological momentary assessment—the process of repeatedly sampling behaviors or experiences in situ in real-time—have been shown to reduce episodic memory decay and recall biases as compared to retrospective questionnaires in patients with high-functioning autism [53]. Moreover, responses on mobile apps reliably matched standard-of-care questionnaires used in clinical practice, making mobile symptom and side-effect tracking a promising option for ASD patients [53]. Though real-time data-driven apps that are used by patients (as opposed to their parents) have the limitation of being applicable primarily to those with high-functioning ASD, the emergence of mobile technologies that cater to patients with ASD that have lower cognitive function continue to prove promising [55].

Phone call and text interventions in adolescent mental health, and especially in adolescent depression and ASD, offer promising ways to improve care, with unique advantages over other digital interventions such as teleconferencing or mobile apps. First, while access to videoconferencing and mobile devices may be limited, phone access is ubiquitous, with more than 90% of the population having access to a mobile phone [56]. Therefore, phone calls and text messaging offer inexpensive alternatives to apps requiring mobile phones with app capabilities (smartphone) or standalone devices. Second, automated texting and calling platforms allow proactive prompting, which improves engagement over mobile apps and allows highly resolved treatment, symptom, and side effect tracking. Third, texting is the primary mode of communication for adolescents, making text-delivered interventions a natural extension of communication with patients’ healthcare providers. As such, we set out to build text message interventions for adolescent depression and autism using a patient-centric design process that addressed some of the major pain points in the field.

## Methods

Epharmix is an automated texting and calling platform that sends standardized, condition-specific texts or calls to patients or their designated caregivers to track symptoms longitudinally in real-time, provide educational content to patients, and trigger smart alerts to providers when patients report concerning symptoms or behaviors. Two specific interventions for depression and ASD, called EpxDepression and EpxAutism, respectively, have been developed. EpxDepression and EpxAutism were developed using a human-centric design methodology that involved identifying and interviewing seasoned clinicians, research scientists, and biostatisticians in adolescent psychiatry, conducting an extensive literature review, developing optimal questions, alert thresholds, and a clinical decision tree (together known as the “algorithm”), running a small-scale pilot study in a clinically relevant adolescent population as a method for validating algorithm parameters, analyzing data and iterate on the algorithm, using both user feedback and continued research, and interfacing with seasoned clinicians. The unique iteration and development cycle allows rapid, data-driven improvements of the Epharmix systems. Currently, we have run a small pilot for both EpxDepression and EpxAutism and are updating each algorithm based on our results, as well as feedback from providers and patients. These implementations were submitted to the institutional review board (IRB) for review and advised to be pursued as quality improvement (QI) projects. Consent for these studies was received from patients via the Washington University in St Louis “Authorization to utilize unencrypted email/ text messages to communicate protected health information” form as well as verbal consent from patients in accordance with their provider.

### EpxDepression

**Figure 1:**
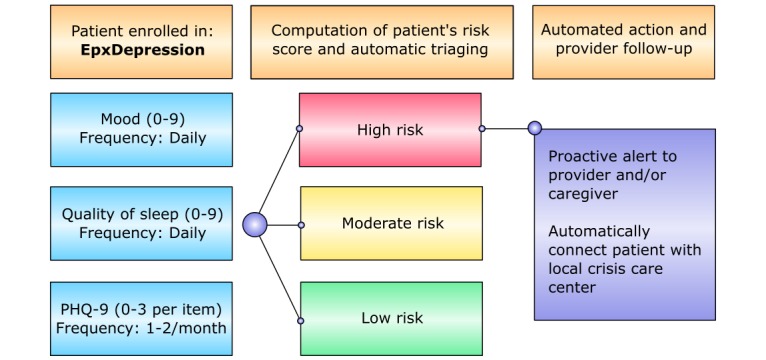
EpxDepression automated text message algorithm.

EpxDepression prompts the patient or caregiver for subjective mood and sleep ratings on a 10-point rating scale every day, and sends a text-based modified PHQ-9 every 2 weeks or month, depending on the provider’s assessment of the patient (Figure 1). While the modified PHQ-9 is administered through a text message platform as opposed to in-person, its scoring function was adapted to give an equivalent score to the original PHQ-9. Depending on the patient’s mood, PHQ-9 score, and self-harm ideation (a positive response on the last question of the PHQ-9), the patient is triaged into a red-yellow-green hierarchy. Patients who are scored as high-risk (red) are automatically reported to the care team. Importantly, patients who report self-harm behavior are automatically directed to a suicide help-line, crisis manager, or entrusted caregiver (Figure 1).

In our initial pilot study with EpxDepression, our primary aim was to assess patient engagement with an automated texting platform. We enrolled 3 adolescent patients with MDD in EpxDepression and assessed their weekly response rates to the mood, sleep, and PHQ-9 prompts. A weekly response is defined as having answered at least 1 question during the week for the associated prompt. The frequency of the PHQ-9 questionnaire was determined by the specific program the patient was placed in (once every 2 weeks or once a month), while the mood and sleep questions were asked daily. The duration of enrollment varied on a case-by-case basis, spanning anywhere from 2 to 4 months.

### EpxAutism

**Figure 2:**
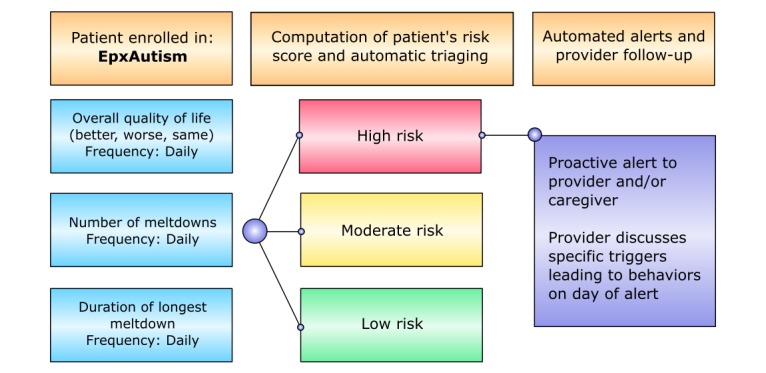
EpxAutism automated text message algorithm.

In our initial pilot with EpxAutism, 6 adolescent patients with ASD and their parents were enrolled in the study. The duration of enrollment varied on a case-by-case basis, spanning anywhere from 18 to 110 days. EpxAutism prompted each patient’s caregiver daily for the patient’s quality of life, the number of meltdowns the patient has had in the last 24 hours, and the duration of the longest meltdown (Figure 2). The system synthesized data received through text responses for each individual and triaged patients into 1 of 3 risk categories including red (high risk), yellow (moderate risk), and green (low risk) based on a change in meltdown frequency and duration as set by their provider and sent alerts to providers upon recognizing significant upward deviations in recorded meltdowns.

The aim of EpxAutism was to improve a provider’s ability to monitor patient behavior and symptoms between visits, foster communication between the patient and provider, and improve survey compliance and health outcomes for the patient. Our primary outcome was weekly response rates, but we were also interested in overall response rates, behavioral metrics reported by the caregivers, and feedback from parents using the system. Similar to our pilot study with EpxDepression, weekly response was defined as having answered at least 1 question during the week for the associated prompt, and overall response rate was defined as the number of prompts answered divided by the number of prompts sent to the parent.

**Survey prompts.**

Prompt

- Communication: On a scale of 1 to 9, do you think this service improved communication with your doctor? (1 = significantly worsened, 5 = no change, 9 = significantly improved).
- Frequency: On a scale of 1 to 9, how do you feel about the number of messages you received through our service? (1 = too few, 5 = perfect amount, 9 = too many).
- Improvement: How could we make this service better? Feel free to write as much as you would like.
- Positive Comment: What did you like about this service? Feel free to write as much as you would like.

### User Feedback

Users were sent monthly surveys through text messages which asked for subjective feedback on the EpxDepression or EpxAutism systems. Deidentified survey data was used frequently to iterate on specific questions, timing of messages, and triaging methodology. Prompts within the survey are shown in Textbox 1.

## Results

### EpxDepression

**Figure 3:**
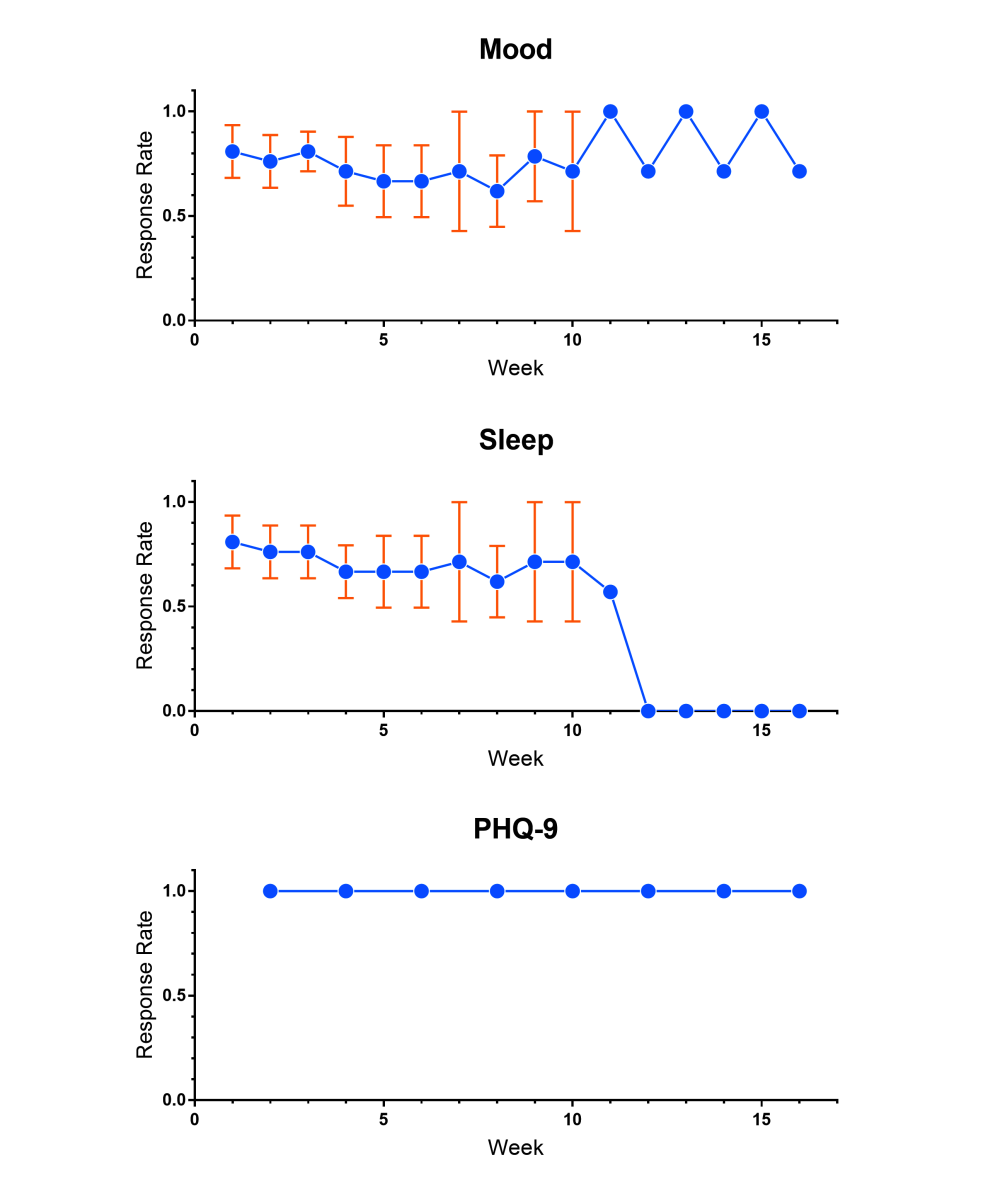
EpxDepression patient overall response rates.

We found that with the mood, sleep, and PHQ-9 questionnaires, patients maintained a 100% weekly response rate during their enrollment in the study, indicating that no patients were lost to follow-up. However, overall response rates for mood and sleep, defined as the percentage of questionnaires completed for mood, sleep, or PHQ-9 whenever prompted, was markedly lower (Figure 3). The mean (SD) daily response rate for mood was 66.3% (21.6%) and sleep was 64.7% (8.2%). The response rates decreased over time, with the sleep prompt having lower response rates and greater decay than the mood prompt from the outset (Figure 3). Our preliminary data suggested that adolescents with MDD engaged with prompts on a weekly basis in a robust fashion, but underwent message fatigue with daily prompts. A larger study will be necessary to confirm our findings.

**Figure 4:**
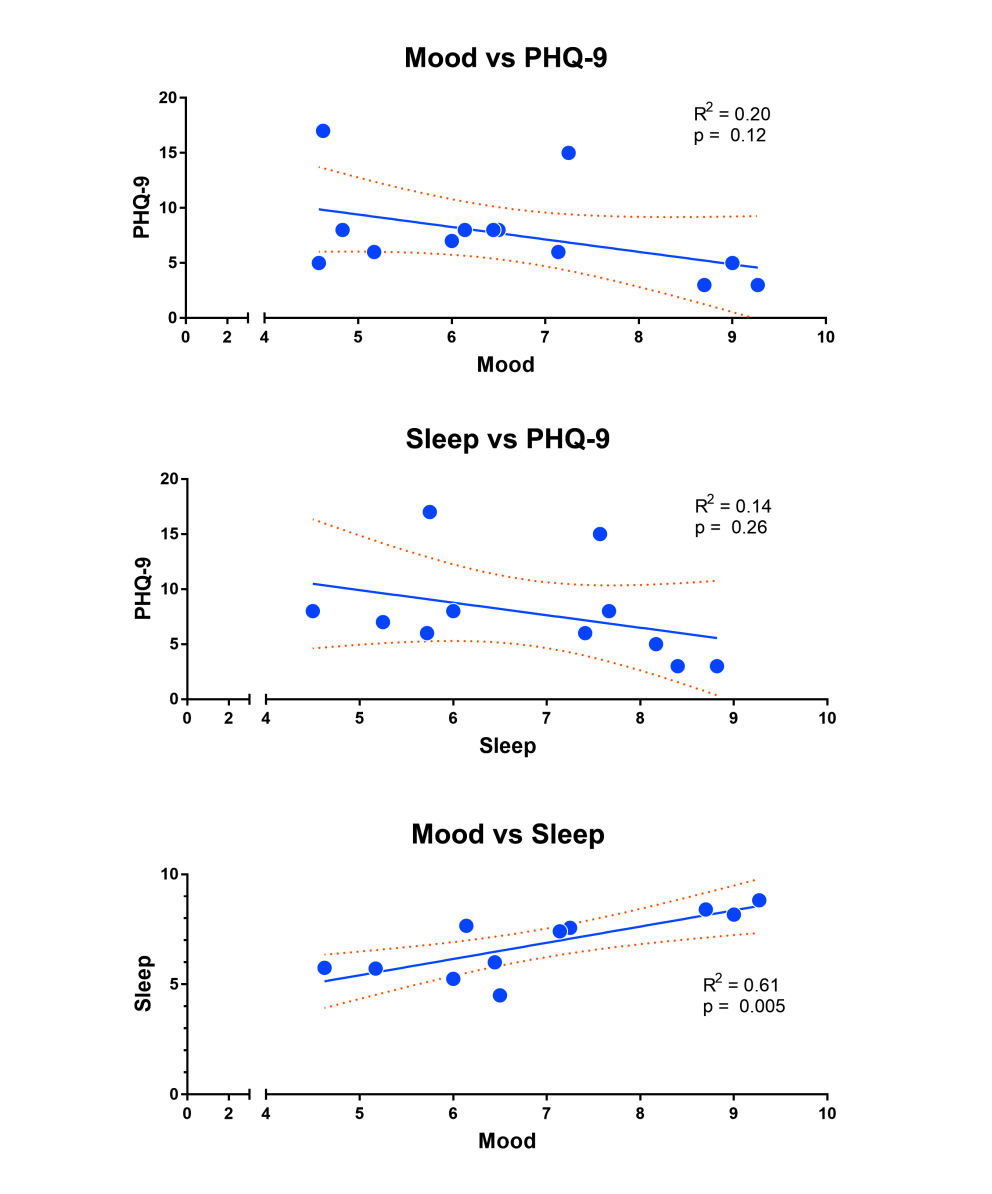
EpxDepression correlations between outcomes.

While our primary outcome was weekly response rates over time, we wanted to determine whether self-reported mood or sleep ratings could predict a patient’s PHQ-9 score as suggested in other studies [34]. We averaged each patient’s mood or sleep ratings leading up to a PHQ-9 prompt, and regressed these averages against the patient’s PHQ-9 score for that time point. Based on our data, mood (R^2^=.20, *P*=.12) and sleep (R^2^=.14, *P*=.26) were not significantly correlated to the PHQ-9 score, although both trended toward a negative correlation as might be expected (Figure 4). Self-reported mood ratings were positively correlated with sleep ratings (R^2^=.61, *P*=.005) and consistent with the literature (Figure 4). Due to the small sample size, these results are suggestive; future studies with a larger sample size will be needed to determine whether self-reported mood and sleep ratings can predict self-reported PHQ-9 scores collected through automated text-messaging.

Despite the small sample size, the EpxDepression module has already made a tangible impact in the lives of our patients. EpxDepression is programmed to automatically alert the patient’s care provider if the patient reports a positive response to the suicidal ideation question (question 9) on the PHQ-9 survey. In the 2 patients who scored positively on this question, EpxDepression detected and alerted the patients’ care providers of the patients’ self-harm ideations, even before the case manager was aware that the patient was having these thoughts. The case manager was able to follow-up with each patient and confirm that the patient was having suicidal ideation, and care was modified appropriately. These instances demonstrated that patients with MDD in our study were willing to disclose suicidal ideation through text-messaging before telling their case manager or families in person.

From adolescent patients enrolled in EpxDepression, 7 feedback surveys were completed where users answered a valid response for at least 1 of the prompts. From the small sample size, users indicated that the number of messages received was the “perfect amount” (median=5) where 1 was too few, 5 was the perfect amount, and 10 was too many. There was no improvement in communication between patient and provider attributed to the intervention, as observed by a mean value of 5, where 1 indicated significantly worsened, 5 indicated no change, and 9 indicated significantly improved. Positive feedback commonly included improved feeling of support and contact between patient and provider:

“Knowing my Dr [sic] is in contact and aware of my answers.”

“I like the daily contact.”

“Service gave me feeling of daily contact with my Dr [sic].”

Despite indicating that there may not have been any improvement in communication with providers, no patient provided any negative feedback based on survey results. Most feedback included confirmation of “service works fine” or “service is great” when queried for areas of improvement.

### EpxAutism

**Figure 5:**
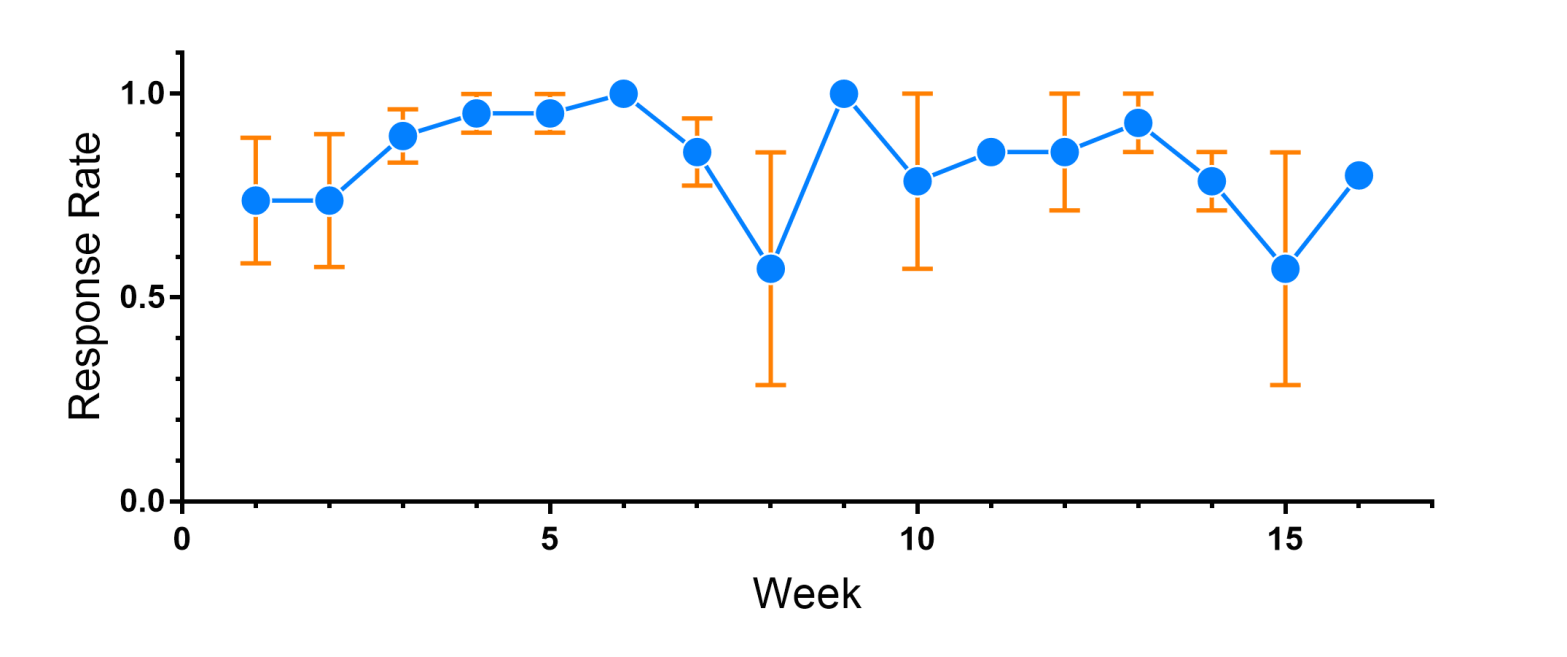
EpxAutism patient overall response rate.

The weekly response rate for parents using EpxAutism was 100%, indicating a robust and prolonged enrollment in the system that did not decay with time. Remarkably, the mean (SD) overall response rate even with daily questions was 85% (29.1%) (Figure 5). Unlike with mood and sleep prompts in EpxDepression, there was no significant difference between overall response rates between individual prompts in EpxAutism. High engagement rates were maintained throughout the duration of the pilot. Our data suggested that EpxAutism is an engaging system for caregivers with children with ASD and may be used to better track concerning symptoms and behaviors on a daily basis.

Participants enrolled in EpxAutism completed 18 surveys in which a valid response for at least 1 of the prompts was submitted. When queried about communication between patient and providers, users responded to “On a scale of 1 to 9, do you think this service improved communication with your doctor?” On this scale, 1 indicated significantly worsened, 5 indicated no change, and 9 indicated significantly improved. The determined median score of 7 indicated an improvement in communication from use of EpxAutism.

In terms of frequency of text messages, parents received 3 prompts daily about their child’s behavior. When surveyed, parents responded to “On a scale of 1 to 9, how do you feel about the number of messages you received through our service? On this scale, 1 indicated too few, 5 indicated the perfect amount, and 9 indicated too many. Patients reported a median response value of 5.

Positive comments often reflected the importance of daily communication and feeling connected with their provider:

“Daily contact and reporting is what we need to help identify issues and help our son.”

“More communication is better and daily tracking is needed. Thanks.”

“The service is great. I appreciate all that [provider] has done to teach [my son] to better control himself and he has helped me with ways to help my son as well. I liked having a record of [my son’s] improvement. It seemed to help [my son] knowing [provider] was checking up daily to see how well he was doing.”

Areas of improvement almost universally revolved around caregiver desire to input more information to providers:

“Be able to provide [sic] a few more details.”

“I think a comment space would be nice.”

”Allow for additional comments about behaviors...I think it would be helpful to include additional commentary about time of day or specific issues.“

## Discussion

### Principal Findings

While pharmacology and psychotherapy have made remarkable progress in addressing patients with adolescent depression and ASD, there exists a dearth of reliable methods to track symptoms over time, monitor behavior, and prevent suicidal behaviors. New ways to leverage mobile technology continue to augment traditional patient monitoring and treatment options. Electronic office visits and CBT sessions have robust literature supporting their efficacy, expanded access to care, time efficiency, and satisfaction among providers and patients alike [5,32,51]. However, there is a much smaller body of literature describing the utility and efficacy of mobile apps, phone call platforms, and text message interventions in adolescent depression and, especially, ASD. Overall, the literature supports mobile platforms as promising ways to deliver and improve adherence to CBT, track symptoms, monitor behavior, and engage patients. Because landline and texting are ubiquitous forms of communication, especially in adolescent populations, these platforms offer a unique angle to improve patient care.

In order to explore text message interventions in adolescent populations with depression and ASD, we developed 2 automated text message systems called EpxDepression and EpxAutism. In our initial pilot with EpxDepression, weekly response rates were very high, whereas overall response rates on a prompt-by-prompt basis suffered from message fatigue. Because only the daily mood and sleep prompts showed signal decay with time, our data suggested that adolescent patients with depression found daily questions about their mood and sleep tiresome, unhelpful to their care, or were simply more engaged with questions asked on the PHQ-9. Patient feedback indicated that frequency of total prompts was satisfactory with no complaints about the mood or sleep questions, suggesting a difference in engagement to these questions attributed to the lack of perceived usefulness or patient interest. As such, we are currently modifying the mood and sleep questions within the EpxDepression algorithm.

Previous studies have shown that subjective mood ratings submitted over the phone correlate with in-person PHQ-9 scores [34]. In our small pilot with EpxDepression, we failed to find a significant correlation between recent mood or sleep scores and PHQ-9 scores. However, given our small sample size and the exploratory nature of this pilot, further investigation will be necessary to determine whether self-reported subjective mood and sleep ratings can predict PHQ-9 scores. In addition, due to the correlation we found between mood and sleep ratings, we plan on incorporating a split testing method for EpxDepression and analyzing response rates with or without sleep prompts, which may help alleviate message fatigue without losing significant amounts of predictive or clinical information.

Perhaps the most illuminating result from the EpxDepression pilot was the capability for a text-based PHQ-9 questionnaire to detect self harm ideation, even before parents and case managers were made aware. Our data is in line with the spontaneous success of text-based hotlines, and may reveal the willingness of adolescents to disclose more personal information about their mental health to electronic devices, which feel less judgmental than face-to-face disclosure [37-39,57]. As such, an inexpensive, proactive, phone-based depression monitoring platform such as EpxDepression may be a valuable tool not only to track mood, sleep, and depressive symptoms between visits, but also to detect suicidal ideation and even prevent suicidal events. We are currently piloting the second iteration of EpxDepression in a larger study to confirm our preliminary findings reported in this pilot.

In our initial pilot with EpxAutism, we found remarkably high weekly and overall response rates that did not appear to decay significantly with time. Though larger studies are needed, if this trend holds true, the difference in overall response rates between our pilot with EpxAutism and our pilot with EpxDepression may be attributed to a difference in user motivation, demographics, or a perceived benefit of each question on the user’s care. Of note, the patient population was particularly different between studies, where users enrolled in EpxAutism were caregivers responsible for their children’s health, whereas EpxDepression was used by adolescents themselves. Future interventions for EpxDepression may be able to utilize the increased caregiver engagement to build complementary monitoring systems, such as a depression intervention for the adolescent patient involving the patient’s parents or caregivers.

Consideration for ways to develop EpxAutism in the future to meet further needs are extensive. Caregiver feedback indicating desire for more input is being examined. Currently, due to feedback from psychiatrists involved in the study, patients are being enrolled who will receive additional daily questions about quantity of meltdowns including dangerous behaviors (specifically self-injurious behavior, aggression, and disruption). Careful consideration will be taken not to drastically increase the number of daily messages and therefore subject users to increased message fatigue. Hand-written daily journals and optional free-text message responses for caregivers are potential solutions for caregivers who wish to provide more information while simultaneously avoiding overwhelming those who do not.

A natural extension to monitoring meltdowns in patients with ASD is empowering families with knowledge and guidance in order to reduce the quantity and duration of such episodes. EpxAutism is exploring using the Epharmix text messaging platform to periodically send caregivers information that teaches them to provide competent applied behavior analysis (ABA) techniques aimed at reinforcing appropriate behaviors and decreasing instances of inappropriate behaviors.

Children with ASD face additional risks that could be addressed via an automated text messaging platform. These children are at increased risk for fractures especially during childhood and adolescent development due to decreased bone density [58]. By monitoring mealtime behavior, dietary intake, physical activity, and screen time (time spent using electronic devices), risk for decreased bone density could be quantitatively measured remotely. In addition, children with ASD have increased rates of gastrointestinal symptoms, as well as changes in satiety and eating habits, compared to children without autism [59]. Thus, monitoring gastrointestinal symptoms, changes in weight, diet, and eating patterns could be a promising way to tailor text-based interventions for children with ASD.

### Conclusion

Phone and text message interventions offer a promising, inexpensive platform to increase access to care for adolescent patients with depression and ASD and give providers a method to track symptoms and behaviors with unprecedented temporal resolution. The unique iteration cycle that we developed with Epharmix enables rapid conversion of feedback from patients and providers into optimized platforms for further testing. We envision EpxDepression and EpxAutism as stepping stones toward replacing traditional behavioral tracking, which is often limited by access and is biased by retrospective completion. While we discussed 2 interventions in this review, we developed a number of additional interventions within the scope of behavioral health and are currently running multiple pilot studies in parallel to confirm their clinical utility. Our goal is to leverage our rapid iteration cycles to develop a fully optimized mobile platform that can be tested in a Phase III RCT. While there are still many hurdles to overcome in adolescent psychiatry, the future standard of care may only be a text message away.

## Abbreviations

ABA: Applied Behavior Analysis
ASD: autism spectrum disorder
CATCH-IT: Competent Adulthood Transition with Cognitive-behavioral Humanistic and Interpersonal Training
CBT: cognitive behavioral therapy
cCBT: computerized cognitive behavioral therapy
CDRS-R: Child Depression Rating Scale Revised
DSM-5: Diagnostic and Statistical Manual of Mental Disorders, Fifth Edition
MDD: major depressive disorder
PHQ-9: Patient Health Questionnaire-9
RCT: randomized controlled trial
